# A One-pot Condensation for Synthesis 2-methyl-4-phenylpyrano[3, 2-c] chromen-5(4H)-one and Synthesis of Warfarin by Ionic Liquid Catalysis

**Published:** 2016

**Authors:** Aazam Monfared, Zohreh Esmaeeli

**Affiliations:** ^*a*^*Department of Chemistry, Payame-Noor University, P. O. Box 19395-3697, Tehran, Iran.*

**Keywords:** Michael addition, Ionic liquid, warfarin, close-ring

## Abstract

The anticoagulant racemic warfarin is synthesized by the Michael addition of 4-hydroxycoumarin with benzalacetone in the present of equimolar amounts of imidazolium based ionic liquids [bmim] BF_4_ and [bmim] Br and other reaction solvents such as H_2_O, pyridine and ammonia in five different tests. Also synthesis of a derivative of warfarin (2-methyl-4-phenyl pyrano [3, 2-c] chromen-5(4H)-one) under solvent-free condition is reported. In this paper, we show the potential that ionic liquid have for the development of green methods for the formation of the C-C bond by reaction condensations without catalysts and organic solvents. A ^،^green method^,^ according to the well-known principles, must reduce or eliminate the use or generation of unsafe substances. The work-up procedures were fairly simple and the products don›t require further purification.

## Introduction

The present article relates to warfarin is known chemically as 4-hydroxy-3-(3-oxo-1-phenyl butyl)-2H-chromen-2-one, and specifically to modified synthesis of it. The most well-known and exploited route to synthesize warfarin involve the direct condensation of 4-hydroxycoumarinwith benzalacetone in pyridine (1), in presence of an ion exchange particularly a poly-(alkylen imine)- resin (2) and alkali substance improved by using alkali metal phosphates (3). Some improved procedures have been reported for the reaction of 3-carbetoxy-4-hydroxycoumarin with benzalacetone and amines in an inert solvent such as H_2_O (4).

In adopting the principles of green chemistry (5, 6), there are two general approaches to organic synthesis: (1) the use of solvent-free and (2) the use of alternative reaction medium such as ionic liquid. Solvent-free reaction protocols used in the condensation reactions such as Michael reactions which are quickly becoming the pre-eminent synthetic approaches (5-7). 

A case report of Pediatric Congenital Hematlogic Disordesrs Research Center of Shahid Beheshid Univ., Tehran, showed that warfarin induced Eosinophilia in a child with Burkitt Lymphoma (8), and also systematic review clinical, 5107 patient with Dabigatran as a direct oral anticoagulants (DOAC) for treatment of acute VTE (Venous Thromboembolism), showed in minor bleeding-the Dabigatran seemed as effective as, but at last mentioned, new researches are needed to be clarified (9). 

Ionic liquids, which have negligible vapor pressure, have become commonplace in research laboratories as variable reaction solvent alternative to volatile organic solvents (10-12). In this paper, we show the potential that ionic liquid have for the development of green methods for the formation of the C-C bond by reaction condensations without catalysts and organic solvents. A ^،^green method^,^ according to the well-known principles, must reduce or eliminate the use or generation of unsafe substances. As part of our continuing interest to develop more efficient and environmentally benign methods for organic synthesis using eco-friendly materials as catalysts [13], herein we describe an efficient synthesis of warfarin from 4-hydroxycoumarin with benzalacetone in Michael reaction in [bmim] BF_4_ and [bmim] Br and the synthesis a derivative of warfarin under solvent-free condition. 

**Table 1:** Michael reaction of 1 and 2 in different reaction solvent to form warfarin 3 and its derivative 4.

**Entry**	**Reaction solvent**	**Reaction temperature (** ^°^ **C)**	**Reaction time (h)**	**Yield (%)**	**Melting point (** ^°^ **C)**	**Product**
1	[bmim]Br	RT*	5	96	157-160	3
2	[bmim]BF_4_	50	6	82	157-159	3
3	Ammonia & H_2_O	Reflux	4:30	80	155-159	3
4	H_2_O	Reflux	12	57.1	157-160	3
5	Pyridine	Reflux	24	39.4	159-163	3
6	Solvent- free	100	8	75	133-135	4
7	Pyridine(Drop)	Reflux	16	21	130-132	4

* RT: Room Temperature

**Scheme 1:**
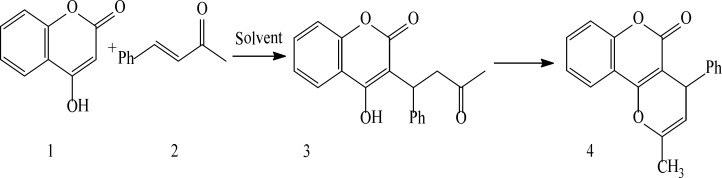
Synthesis of Warfarin and ring-closing derivative of it via a Michael reaction

**Scheme 2:**
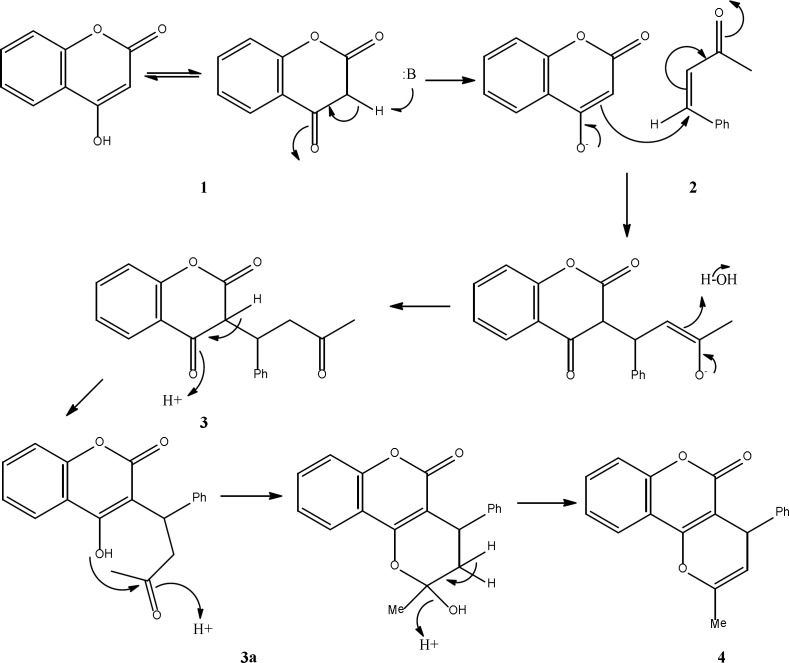
Proposed mechanism for the formation of compounds 3 and 4

## Experimental

*Chemical and apparatus*

4-Hydroxycoumarin, benzalacetone and [bmim] BF_4_ has been bought from *Merck* and was used without further purification. [bmim]Br was synthesized from the reaction of N-methylimidazole and n-butyl bromide (14). Melting point was obtained with an *Electrothermal-9100*apparatus. IR Spectra was recorded with a *Shimadzu IR-21 prestige *spectrometer**. **^1^H and ^13^CNMR Spectra were recorded with a *Bruker BRX-500 Avance* instrument using CDCl_3 _as the deuterated solvent containing tetramethyl silane as internal standard, at 500 and 125 MHz; δ in parts per million, J in hertz. Mass spectra were obtained with a *Finnigan-MAT-8430* mass spectrometer, in m/z.

*General procedures for synthesis of warfarin (* *entries 1-5-* Table 1 *.* *)*

Note: In 5 procedures mentioned below, we used magnet stirred.

1- A mixture of 4-hydroxycoumarin (1, 1 mmol) and benzalacetone (2, 1mmol) and [bmim] Br (1 mmol) were mixed at room temperature for 5 h. Water was added and the resulting product 3 was extracted with ethyl acetate (2x5 mL). The organic phase was dried over anhydride Na_2_SO_4_. And the solvent was evaporated to obtain warfarin 3 in a pure form.

White powder; m. p. 157-160 ^°^C; yield 96%. IR (KBr): 3300, 1680, 1610 cm^-1^. ^1^HNMR: δ= 2.32 (3H, s, CH_3_), 4.22 (2H, d, CH_2_), 4.31 (1H, t, CH), 7.01-7.94 (9H, m, 9CH). ^13^CNMR: δ= 34.8 (Me), 35.8 (CH), 43.1 (CH_2_), 104.6 (C), 117.1 (CH), 124.1 (CH), 124.4 (CH), 127.5 (CH), 128.7 (CH), 129.4 (2CH), 132.0 (2CH), 132.5 (C), 144.1 (C), 153.2 (C), 159.5 (CH), 162.7 (C=O), 200.0 (C=O). EI-MS: m/z (%) = 308 (M^+^, 2), 213 (100), 77 (38). Anal. Calcd for C_19_H_16_O_4_ (308.33): C, 74.01; H, 5.23; O, 20.76%.

2- 4-Hydroxycoumarin (1, 1 mmol) and benzalacetone (2, 1mmol) and [bmim] BF_4_ (1 mmol) was mixed about 8 h at 50 ^°^C. Water (5 ml) was added, and the resulting product 3 was extracted with Ethyl acetate (2.5 mL). The organic phase was dried over anhydride Na_2_SO_4_. And the solvent was evaporated to obtain warfarin 3. Production of compound 3 was characterized by TLC and melting point. 

3- Into a flask equipped with reflux condenser and stirrer, were mixed 4-hydroxycoumarin (1, 5 gr), benzalacetone (2, 5 gr), 35cc H_2_O and 0.11cc ammonia. Then boiled the mixture and maintained at reflux for 2:30 h during this period a heavy precipitate formed. Refluxing was continued for one additional hour with vigorous agitation, and the reaction mixture was cooled to room temperature. The solid crude product was separated by filtration, floated with fresh water, and sucked as dry as possible. The solid crude were suspended in benzene refluxed with stirring for 45 min, cooled, filtered, washed on the filter with fresh benzene and sucked as dry as possible. The solid were dissolved at room temperature in NaOH 5% and the solution was washed three times with CCl_4_.and acidified with strong HCl to pH 1-3. The final product was warfarin. It was filtered off, washed free of chlorides with water and dried.

White powder; m. p. 155-159.4°C; yield 80%.IR (KBr): 3300, 1700, 1640, 1600 cm^-1^. ^1^HNMR: δ= 2.28 (3H, s, Me), 3.33 (2H, d, CH2), 4.17 (1H, t, CH ), 7.18 –7.93 (9H,m, 9CH).^13^CNMR: δ= 30.9 (Me), 36.2 (CH), 46.0 (CH_2_), 104.9 (C), 117.5 (CH), 124.4 (CH), 124.9 (CH), 127.4 (CH), 128.8 (CH), 129.5 (2CH), 130.0 (2CH), 133.3 (C), 143.9 (C), 154.6 (C), 162.6 (CH), 167.2(C=O), 213.0 (C=O). 

4- Into a flask equipped with reflux condenser and stirrer, were charged with 1 (1 mmol), 2 (1 mmol) and H_2_O. The mixture was heated to boiling with stirring and it formed at reflux for 12 h and the reaction mixture was cooled to 0 °C one overnight. A heavy gum formed. The aqueous phase removed by decantation and recrystallized from an acetone-water mixture. 

White powder; m. p. 157-160 °C; yield 57.1%.IR (KBr): 3300, 1680, 1610 cm^-1^.^ 1^HNMR: δ= 2.27 (3H, s, Me), 3.30 (2H, d, CH_2_), 4.15 (1H, t, CH), 7.11-7.93 (9H, m, 9CH).^ 13^CNMR: δ= 30.8 (Me), 36.1 (CH), 45.9 (CH_2_), 105.0 (C), 117.4 (CH), 124.4 (CH), 124.7 (CH), 127.3 (CH), 128.7 (CH), 129.4 (2CH), 130.0 (2CH), 132.8 (C), 144.0 (C), 153.7 (C), 159.7 (CH), 162.2 (C=O), 212.0 (C=O).

5- Into a flask equipped with reflux condenser and stirrer, are charged with 1 (1 mmol), 2 (1 mmol) and pyridine (as solvent and catalyst). The mixture was heated to boil with stirring and maintained at reflux for 24 h. Through this process, a heavy gum formed, and the reaction mixture was cooled at room temperature. After which it was poured into about 15 volumes of water, and acidified to about pH 2 by the addition of HCl concentrate. The reaction mixture was cooled to 0 °C one over night. The solid recovered with filtration, and recrystallized from ethanol. 

White powder; m. p. 159-163 °C; yield 39.4%.IR (KBr): 3300, 1680, 1600 cm^-1^.^ 1^HNMR: δ= 2.33 (3H, s, Me), 3.37 (2H, d, CH_2_), 4.21 (1H, t, CH), 7.18-7.79 (9H, m, 9CH).^ 13^CNMR: δ= 30.5 (Me), 35.8 (CH), 43.0 (CH_2_), 104.6 (C), 117.1 (CH), 124.0 (CH), 124.3 (CH), 127.4 (CH), 128.6 (CH), 129.6 (2CH), 131.9 (2CH), 132.4 (C), 143.6 (C), 153.4 (C), 159.2 (CH), 162.5 (C=O), 218.6 (C=O).

*General procedures for synthesis of compound 4; 2-methyl-4-phenyl pyrano [3, 2-c] chromen-5(4H)-one (* *entries 6-7* Table 1 *.)*

6- Into a tube equipped with stirrer, are mixed about4-hydroxycoumarin (1, 2.5 mmol) and benzalacetone (2, 2.5 mmol) and it was placed into oil bath, stirred for 8 h and the reaction mixture was cooled to 0 °C one over night. A heavy gum formed that was 4 and it was recrystallized from ethanol. 

White powder; m.p. 133-135 ^°^C; yield: 75%. IR (KBr): 1725, 1640, 1600-1520 cm^-1^. ^1^HNMR:δ= 2.06 (3H, s, CH3), 4.49 (1H, d,^3^*J* 4.3, CH), 5.04 (1H, d, ^3^*J* 4.3, CH), 7.18-7.87 (9H, m, 9 CH). ^13^CNMR: δ= 18.8 (Me), 36.7 (CH), 103.5 (CH), 104.1 (C), 114.6 (C), 116.8 (C), 122.9 (2CH), 124.2 (C), 127.2 (2CH), 128.4 (C), 128.7 (CH), 129.3 (CH), 132.0 (CH), 144.4(C), 146.2 (CH), 152.9 (C), 161.8 (C=O).EI-MS: m/z (%)= 290 (M^+^, 32), 289 (99),Anal. Calcd for C_19_H_14_O_3_ (290.31): C, 78.61; H, 4.86; O, 16.53%.

7- Into a three-neck flask, equipped with reflux condenser and stirrer, were dissolved about 1 (1 mmol) and pyridine (solution I) and in a dropping tube were dissolved 2 (1 mmol) benzalacetone and pyridine (solution II), (I) it was maintained at reflux for 16 h and in different time interval was trickled several droplets of solution II. Then the solution was cooled and was added about 15 volumes of water, and was acidified to about pH 2 through adding HCl. Oil was separated, and then cooled to 0 °C one overnight. The solid recovered as by filtration, and recrystallized from ethanol as compound 4.

White powder; m.p. 130-132 ^°^C; yield: 21%. IR (KBr): 1720, 1640, 1620-1560 cm^-1^. ^1^HNMR:δ= 2.07 (3H, s, Me), 4.48(1H, d,^3^*J* 4.0, CH), 5.06 (1H, d, ^3^*J* 4.0, CH), 7.16-7.85 (9H, m, 9 CH). ^13^CNMR: δ= 18.6 (Me), 36.4 (CH), 103.4 (CH), 103.9 (C), 114.4 (C), 116.7 (CH), 122.7 (2CH), 124.0 (C), 127.0 (2CH), 128.2 (C), 128.5 (CH), 128.9 (CH), 131.8 (CH), 144.2 (C), 146.0 (CH), 152.7 (C), 161.6 (C=O).

## Results and discussion

In this paper, we examined the efficiency of different reaction solvents for Michael condensations of 4-hydroxycoumarin1 and benzalacetone2. (scheme1). Significant rate improved yields were observed using solvent- free condition and Room-Temperature Ionic Liquids (RTILs) as reaction mediator. (Table 1.)

The results showed that the RTIL [bmim]Br was the best reaction solvent in terms of yields and reaction times for synthesis 3. The reaction of 4-hydroxycoumarin, benzalacetone and ammonia as catalyst and water as solvent required 4:30 h under refluxing temperature and yielded 80%, while in [bmim] BF_4_, an 82% yield of the same product was obtained in 6 h at 50 ^°^C. 

The reaction of 1 and 2 and H_2_O as solvent was achievable and required 12 h under refluxing temperature and yielded 57.1% while in pyridine as catalyst and solvent, the yield was 39.4% in 24 h and under refluxing temperature. Subsequently, we investigated the scope of the condensation reaction of 4-hydroxycoumarin, benzalacetone in [bmim] Br for synthesis of warfarin. All the products were characterized by ^1^HNMR and ^13^CNMR Spectra, IR, Mass spectrometry and melting point. For every reaction 1 and 2, the ionic liquid was recovered by extraction, followed by evaporation from water and washing with ethyl acetate and drying with Na_2_SO_4_. After the evaporation of ethyl acetate, the ionic liquid could be reused several times without any loss of activity. The use of room temperature ionic liquids (RTILs) in this reaction reduced the time of reaction.

In the entry 6, the reaction of 4-hydroxycoumarin1, benzalacetone2 was carried out under solvent- free condition and required 8 h, the yield was 75%, while in the entry 7, the product was obtained under refluxing temperature and yielded 21% that the product of both of them was 2-methyl-4-phenyl pyrano [3, 2-c] chromen-5(4H)-one4 that was characterized by ^1^HNMR and ^13^CNMR spectra, IR, Mass Spectrometry and melting point.

A tentative mechanism for this transformation is proposed in Scheme 2. Apparently, the reaction proceeds step by step to generate the product 3 which the intermediate 3a, eliminate H_2_O to produce final product 4.

## Conclusions

In conclusion, we have studied Michael addition for synthesis of warfarin and its ring-closing derivative and investigated the influence of reaction conditions on the yield and the kind of product. We have developed an efficient, convenient method for synthesis of warfarin by using ionic liquid as a new solvent that reaction time was reduced, and yield was improved by this new method. The works up procedures were fairly simple and products didn›t require further purification.
